# Comparative analysis of differentially expressed genes and transcripts in the ovary of yak in estrus and anestrus

**DOI:** 10.1080/10495398.2024.2427757

**Published:** 2024-11-18

**Authors:** Chongfa Yang, Yahua Yang, Bingzhu Zhao, Enyu Gao, Hao Chen, Yang Li, Junyuan Ma, Jine Wang, Songming Hu, Xiaochen Song, Ying Chen, Gengsacairang Yang, Shengdong Huo, Wenxue Luo

**Affiliations:** a College of Life Sciences and Engineering, Northwest Minzu University, Lanzhou, Gansu, China; b Gannan Prefecture Animal Husbandry Technology Service Center, Gannan, Gansu, China; c Tianzhu County Animal Husbandry Technology Extension Station, Wuwei, Gansu, China

**Keywords:** Estrus, Anestrus, Omics, Yak

## Abstract

Since most yaks have a long postpartum anestrus period, postpartum anestrus is the main factor affecting the reproductive efficiency of yaks. In this study, the third-generation sequencing technology was used to successfully screen differentially expressed genes (DEGs) and differentially expressed transcripts (DETs) in the ovarian tissues of yaks during estrus and anestrus. The functional references of DEGs and DETs were Gene Ontology, Kyoto Encyclopedia of Genes and Genomes, and Clusters of Orthologous Genes database. A total of 1149 DEGs and 2294 DETs were successfully identified. These DEGs and DETs were mainly related to biological processes such as “reproduction”, “reproductive process”, “metabolic process” and “rhythmic process”. Kisspeptin-G protein-coupled receptor was found to be involved in regulating the reproductive cycle of yaks. DEGs and DETs were also related to gonadotropin-releasing hormone (GnRH) signaling pathways such as oocyte meiosis, estrogen signaling pathway, and progesterone-mediated induced oocyte maturation. The results showed that *SIRT1*, *CSNK1A1*, *SLIT3*, *INHBA*, *INSL3*, *ZP2*, *Clock*, *BMP15*, *Bmal1*, *KISS1*, and *LCHGR* regulate the postpartum quiescent state and the reproductive cycle of yaks. This study will help to further clarify the reproductive mechanism of yaks at the molecular level and provide certain assistance for the development of animal husbandry.

## Introduction

The domestic yak (*Bos grunniens*) is a large, long-haired cattle native to the Himalayas and an important working animal for farmers on the Tibetan Plateau (1, 2). In addition, yaks are a valuable genetic resource with important ecological and social value (3). However, the reproductive capacity of yaks is very low. The average natural reproduction rate of female cows is only about 48%, and calves are born once every 2-3 years (4). The yak’s breeding season is seasonal, with female yaks entering a 21-day estrus cycle from July to September. Most female yaks experience ovarian cycle suppression after giving birth, also known as postpartum anestrus. Factors that trigger postpartum anestrus in yaks include nutritional status (5) and climatic conditions (6), Although the key biological factors and underlying mechanisms remain unclear.

Genomics is a relatively new discipline that studies the structure and function of an organism’s entire set of genetic material (7), while transcriptomics is the study of cellular gene transcription and transcriptional regulation at the RNA level (8). Various high-throughput gene sequencing methods have been developed to study disease mechanisms and identify therapeutic biomarkers (9). As a third-generation sequencing technology, Oxford Nanopore technology uses electrical signals to identify base sequences (10). Lan et al. (11, 12) conducted transcriptome sequencing analysis on yak ovaries based on RNA-Seq technology and found: 6321 new transcripts, 2267 new transcripts were predicted to have the ability to encode proteins, and the GO classification annotations of the new transcripts showed that they were related to reproductive traits. The discovery of new transcripts provides basic data for a deeper understanding of yak reproductive function; Huo et al. (13) combined transcriptomics with quantitative proteomics through RNA sequencing and identified genes and proteins related to postpartum ovarian cycle suppression in female yaks. Zi et al. (14) analyzed the transcriptome differences of yak embryos at different developmental stages by analyzing 2-cell, 4-cell, 8-cell, and morulae produced by IVF, and performed RNA-Seq transcriptome sequencing on blastocysts at five developmental stages. The comparative analysis found that among embryonic transcripts, alternative splicing in the transcription start region and alternative splicing in the transcription termination region accounted for the largest proportion; Xu et al. (15) described the evolutionary characteristics of yak follicle development and identified candidate genes related to the regulation of ovarian status during seasonal reproduction; Pu et al. (16) used RNA-seq technology to analyze the changes in the transcriptome of blastocysts before and after vitrification. The results showed that 9827 and 13567 transcripts were detected in yak blastocysts before and after freezing, respectively. These studies revealed the status of differentially expressed genes in yak embryonic cells at different developmental stages, laying a foundation for the molecular regulatory mechanism of yak development and the improvement of embryo in vitro culture technology. However, there are relatively few studies on differentially expressed genes (DEGs) and differentially expressed transcripts (DETs) in ovarian tissues of yaks during estrus and anestrus.

Therefore, the objectives of this study were to (1) identify DEGs and DETs in ovarian tissues of yaks during estrus and anestrus; and (2) identify key biological factors to further elucidate the molecular regulatory mechanisms of yak reproduction.

## Methods and materials

### Experimental animals and sample collection

This study was approved and monitored by the Animal Experiments Ethical Review Committee of Northwest Minzu University (XBMU-shm-20200012), Lanzhou, China.

In September 2019, 16 healthy female yaks that gave birth in April and were about 5 years old were selected in Haiyan County, Qinghai Province. The female yaks were observed and identified every morning and evening under natural grazing conditions. Male yaks were naturally grazed with the test female yaks while wearing “the teaser cloth”. “the teaser cloth” is a strong, durable, and soft canvas that can withstand the friction caused by the yak’s movement and will not cause discomfort and harm to the yaks. The size can completely cover the male yak’s reproductive organs, and it is not easy to fall off or shift. The shape is rectangular, when fixing, use ropes to tie the teaser cloth firmly to the yak’s waist and abdomen to ensure that the teaser cloth will not be torn, preventing the bull and the cow from mating directly during the estrus detection process. Then observe and identify whether the female yaks are in estrus every day. When the female yaks show strong interest in the male yaks wearing “the teaser cloth”, such as approaching, sniffing, climbing or trying to mate, the female yaks will have an estrus reaction. If the female yaks urinate frequently, the mucus out of the vagina increases, and even a small amount of blood flows out, it can be judged that they are in estrus. Yaks in estrus were used as the control group, and rectal digital examinations were performed twice in a row until October 2019. If the surface of the female yaks’ ovaries was found to be smooth, no large follicles or corpora lutea were found, and the plasma progesterone content was also lower than 0.5 μg/L, it was determined to be postpartum anestrus, and the postpartum anestrus female yaks were used as the experimental group. Finally, 4 yaks in estrus were determined in the control group, and 11 yaks in anestrus were determined in the experimental group. Three yaks were randomly selected from each group, slaughtered by carotid artery bleeding, the abdominal cavity was opened to remove the ovaries and immediately stored in liquid nitrogen.

### RNA extraction, library construction, and RNA sequencing (RNA-seq)

The whole ovary on one side of the yak was used. Total RNA was extracted from each group of pooled ovarian samples using TRIzol reagent (Life Technologies, Carlsbad, California, USA). RNA integrity and concentration were assessed using an Agilent 2100 Bioanalyzer (Agilent Technologies, Inc., Santa Clara, CA, USA). Intact mRNA was isolated using the NEBNext^®^ Poly(A) mRNA Magnetic Separation module (New England Biolabs, Ipswich, MA, USA). According to the manufacturer’s instructions, NEBNext^®^ Ultra^™^ RNA Library Prep Kit for Illumina^®^ (New England Biolabs) and NEBNext^®^ Multiplex Oligos for Illumina^®^ (New England Biolabs) to construct cDNA libraries. The enriched mRNA was fragmented into RNA inserts (∼200 nt) for the synthesis of first- and second-strand cDNAs. Double-stranded cDNA was subjected to end repair/dA tailing and linker ligation. Appropriate fragments were isolated using an Agencourt AMPure^®^ XP DNA Purification system (Beckman Coulter, Inc., Brea, California, USA) and enriched by PCR amplification. The constructed yak cDNA library was then sequenced in single-flow cell mode on the Illumina HiSeq^™^ sequencing platform (Illumina, Inc., San Diego, CA, USA). Transcriptome analysis was performed by mapping the readings to the reference genome. Low-quality readings were identified, including those containing only adapters, more than 5% unknown nucleotides, or Q20 values below 20%. These readings were then aligned to the yak genome using Tophat2 software. The resulting alignment records in BAM/SAM format were further checked to eliminate any potential duplicate molecules.

### Screening and analysis of DEGs

Gene expression levels were calculated using the fragments per million exons mapped (FPKM) method of Cufflinks software (http://cole-trapnell-lab.github.io/cufflinks/). DESeq (https://www.bioconductor.org/packages//2.10/bioc/html/DESeq.html) software was used to identify DEGs in postpartum estrus and anestrus ovaries (17). The raw sequencing data of the six samples are deposited in NCBI Sequence Read Archive (SRA, http://submit. ncbi.nlm.nih.gov/subs/sra) with accession number SRR15880952, SRR15880951, SRR15880950, SRR15880949, SRR15880948 and SRR15880947, respectively. Differences in gene abundance between samples were calculated based on the ratio of fragments per kilobase of transcript per million mapped reads values. Probability (*p*) values were obtained using the Benjamini–Hochberg method to control the false discovery rate (FDR), FDR control method was used to identify the threshold of the p-value in multiple tests to compute the significance of the differences. In this study, only genes with a fold change ≥2 and an FDR significance score ≤ 0.01 were identified as DEGs (18). The DEGs were annotated and enriched in reference to the Gene Ontology (GO) (https://geneontology.org/), Kyoto Encyclopedia of Genes and Genomes (KEGG) (https://www.kegg.jp/kegg/), and Clusters of Orthologous Genes (COG) (http://www.ncbi.nlm.nih.gov/COG/) databases using a significance threshold of *p* < 0.01.

### Screening and analysis of DETs

Paired comparisons were made between the estrus and quiescence groups to identify DETs. The parameters of DETs were correction fold change ≥2 and FDR < 0.01. *p* values were corrected with the use of the Benjamini-Hochberg method. The identified DETs were annotated and enriched with reference to GO, KEGG, and COG databases, with a significance threshold of *p* < 0.01.

### Gene expression analysis by quantitative real-time PCR (qRT-PCR)

Total RNA was extracted from tissues using RNAiso Plus (Takara, Dalian, China), and quality was tested using A260/280(1.9-2.0). Reverse transcription was performed using the RNA PCR Kit (AMV) (TaKaRa, Dalian, China) in a total volume of 10 μL reaction mixture, These included 0.5 μg RNA, 5 mM MgCl_2_, 1 × RT buffer, 1 μL dNTP mixture, 10 U RNase inhibitor, 2.5 U AMV-rt, 1.25 pmol oligo(dT) primer, and 3.75 μL dH_2_O without rase. The cells were incubated at 37 °C for 15 min and 85 °C for 5 s. A real-time PCR assay based on SXBR Premix Ex TaqTM II (Perfect Real Time) (Takara) was developed. The housekeeping gene 18s was used to correct for potential variation in RNA loading. A total of 20 μL of 12.5 μL SXBR Premix Ex TaqTM II (2×), 2 μL of template cDNA, 1 μL of each primer (10 μmol/L), and 4.5 μL of deionized water were used for the amplification reaction. The amplification program consisted of an initial denaturation at 95 °C for 2 min, followed by 40 cycles of denaturation at 95 °C for 10 SEC and annealing and extension at 60 °C for 30 SEC. Fluorescence was measured at the end of each annealing and extension step. The cycle threshold (Ct) value was restored to baseline at each reaction, and the presence of specific products or primer dimers in the PCR at each reaction was determined from the melting curve.

## Results

### Identification of DEGs and DETs between the estrus and anestrus periods

After FDR screening, 1149 DEGs (523 up-regulated and 626 down-regulated) and 2294 DETs (1084 up-regulated and 1210 down-regulated) were identified in anestrous ovaries compared with estrous ovaries.

### GO annotation and enrichment analysis of DEGs and DETs

**Figure 1.**
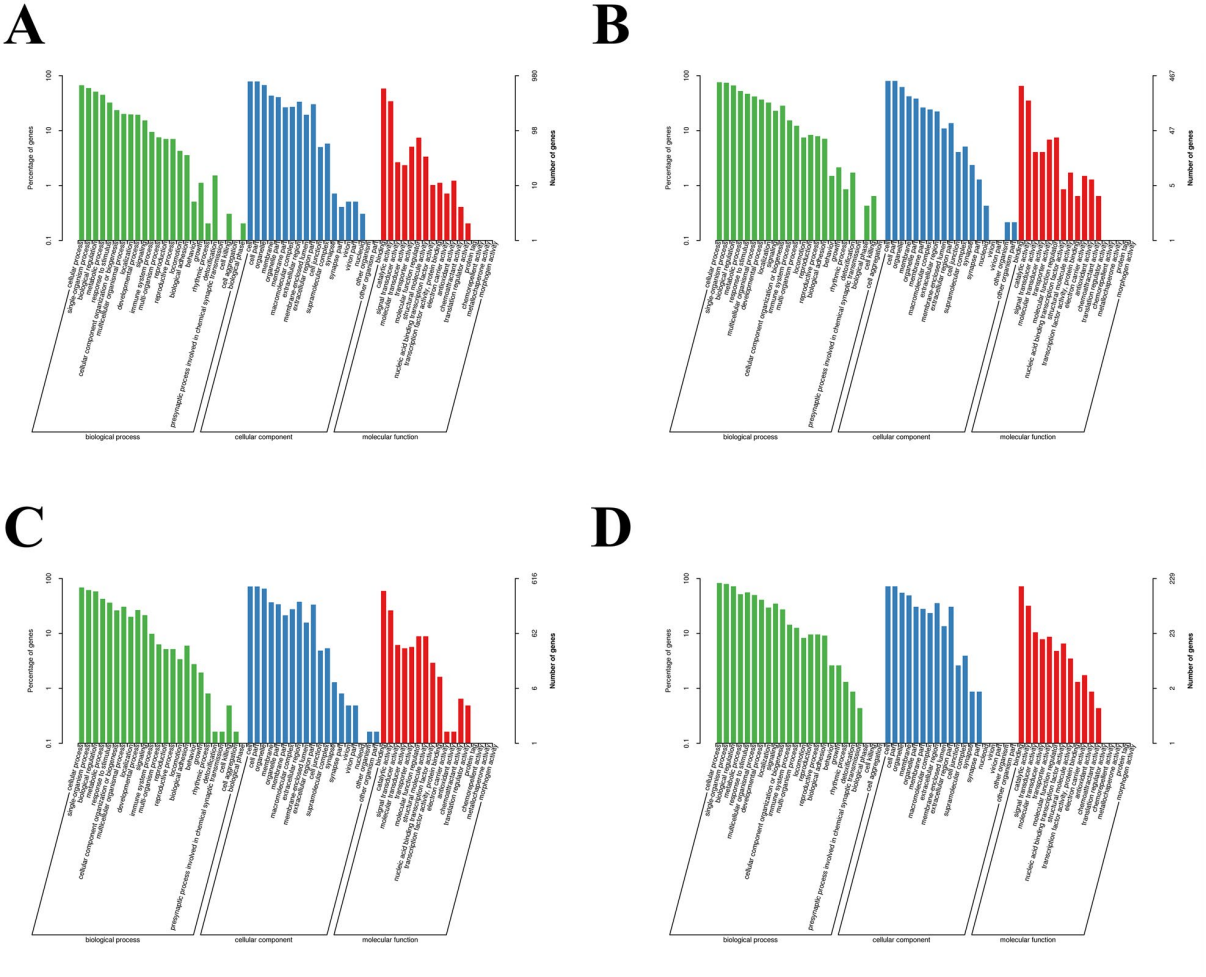
The estrus period served as the control group, and the GO enrichment levels of DEGs and DETs in yak ovaries during the anestrus period were analyzed. (A) Enrichment level of DEGs up-regulated in GO in yaks during anestrus compared with estrus. (B) Enrichment level of DEGs down-regulated in GO in yaks during anestrus compared with estrus. (C) Enrichment levels of DETs up-regulated in yaks during anestrus compared with estrus. (D) Enrichment levels of DETs in GO of yaks with down-regulated expression during anestrus compared with estrus. GO, Gene Ontology; DEG, differentially expressed gene; DET, differentially expressed transcript.

GO analysis was performed to determine the various cellular components, biological processes, and molecular functions in which DEGs and DETs were involved. Compared with the estrus period, the upregulated DEGs and DETs in the quiescent period were mainly distributed in 43 and 49 GO functional terms (Figures 1A and C), while the downregulated DEGs and DETs were mainly distributed in 48 and 49 GO functional terms (Figures 1B and D). Enrichment analysis showed that DEGs and DETs were mainly enriched in biological processes such as “reproduction”, “reproductive process”, and “rhythmic process”. GO terms related to “reproduction”, “reproductive process”, “metabolic process”, and “rhythmic process” in yaks were selected for further analysis. In the biological process category, the most enriched functional items were “cellular process”, “single biological process”, “regulation of biological process”, and “metabolic process”. A total of 120 DEGs (44 upregulated, 76 downregulated) and 202 DETs (64 upregulated, 138 downregulated) were related to “reproduction” and “reproductive process”. In addition, 365 DEGs (119 up-regulated, 246 down-regulated) and 705 DETs (264 up-regulated, 441 down-regulated) were related to “metabolic process”. There were also 7 DEGs and DETs related to “rhythmic process”. In the cellular component category, the most enriched functional items were “cell”, “cell part”, and “organelle”. The DEGs and DETs enriched in the molecular function category were mostly related to “enzyme activity” and “binding”. In total, 378 DEGs (73 up-regulated, 305 down-regulated) were related to “binding”, while 330 DEGs (165 up-regulated, 165 down-regulated) were related to “catalytic activity”. In addition, 43 DEGs (24 up-regulated, 19 down-regulated) were related to “signal sensor activity”. In addition, 940 DETs (367 up-regulated and 573 down-regulated) were associated with “binding”, 497 DETs (163 up-regulated and 335 down-regulated) were associated with “catalytic activity”, and 64 DETs (38 up-regulated and 26 down-regulated) were associated with “signal sensor activity”.

### KEGG annotation and enrichment analysis of DEGs and DETs

**Figure 2.**
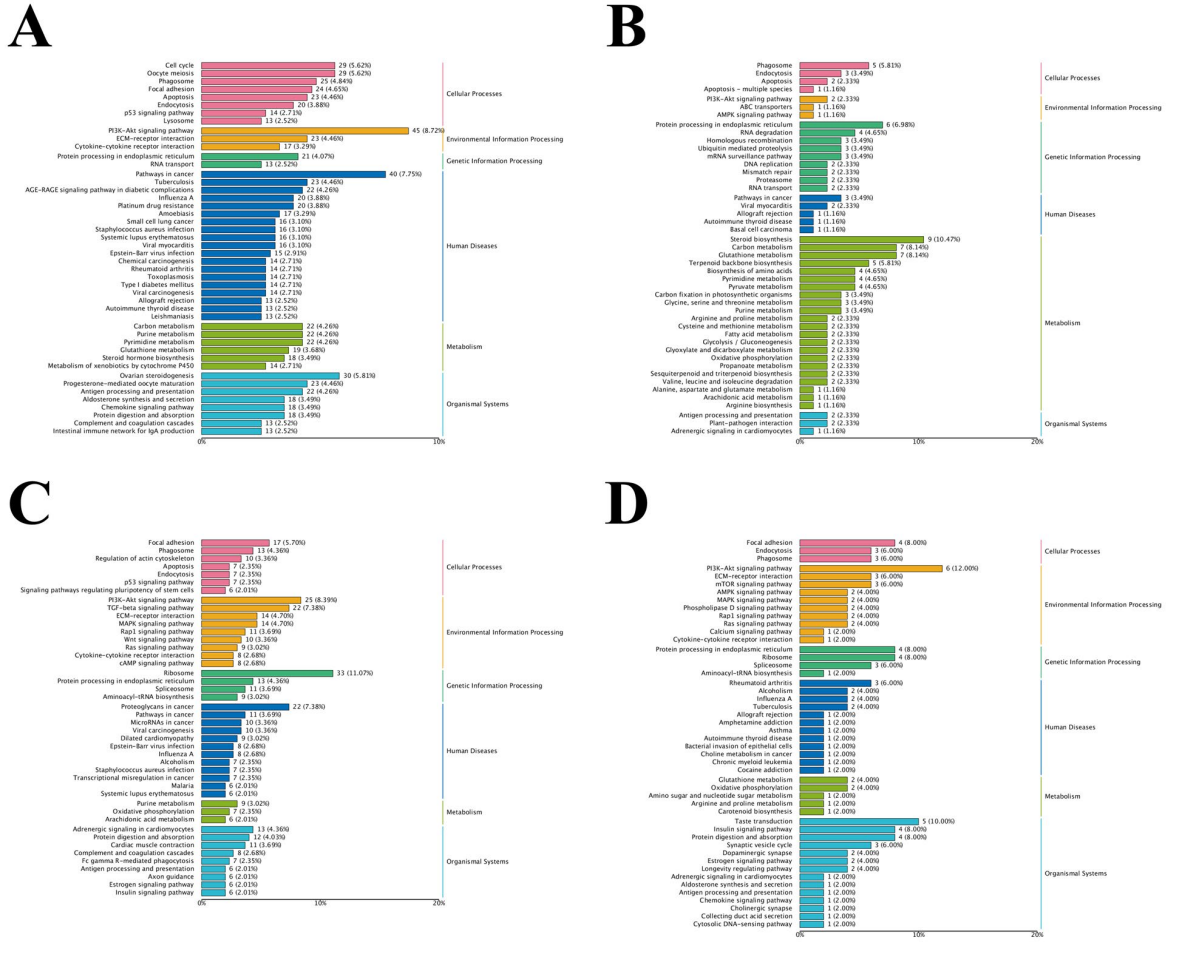
The estrus period served as the control group, and the KEGG enrichment levels of DEGs and DETs in yak ovaries during the anestrus period were analyzed. (A) Enrichment level of DEGs up-regulated in KEGG in yaks during anestrus compared with estrus. (B) Enrichment level of DEGs down-regulated in KEGG in yaks during anestrus compared with estrus. (C) Enrichment level of DETs up-regulated in KEGG in yaks during anestrus compared with estrus. (D) Enrichment level of DETs down-regulated in KEGG in yaks during anestrus compared with estrus. KEGG, Kyoto Encyclopedia of Genes and Genomes; DEG, differentially expressed gene; DET, differentially expressed transcript.

The KEGG database can systematically analyze gene functions and link genomic information with high-order functional information. Pathway analysis can detect DEGs and DETs related to biochemical metabolism and signal transduction. Based on KEGG analysis (Figures 2A-2D), compared with the estrus period, the DEGs and DETs in the ovarian estrus period were mainly related to “cellular processes”, “environmental information processing”, “genetic information processing”, “metabolism”, and “biological systems”. A total of 82 upregulated DEGs were associated with 36 signaling pathways, of which 29 DEGs were enriched in “biological systems”, accounting for ∼35.4%. At the same time, 117 downregulated DEGs were associated with 41 signaling pathways, of which “metabolism” was the most enriched category. In addition, 351 upregulated DETs were associated with 32 signaling pathways, and 568 downregulated DETs were associated with 27 signaling pathways. Signaling pathways related to the regulation of reproductive hormone secretion include “ovarian steroidogenesis” and “steroid hormone biosynthesis”, and signaling pathways related to energy metabolism include “amino acid biosynthesis”, “carbon metabolism” and “glutathione synthesis”. Other signaling pathways related to follicular development include the PI3K-Akt-mTOR signaling pathway, which is also related to follicular development. KEGG analysis of DEGs and DETs found that postpartum estrus in yaks is closely related to sex hormone secretion disorders, nutritional deficiencies or energy metabolism imbalances, and abnormal follicular development.

### COG annotation and enrichment analysis of DEGs and DETs

**Figure 3.**
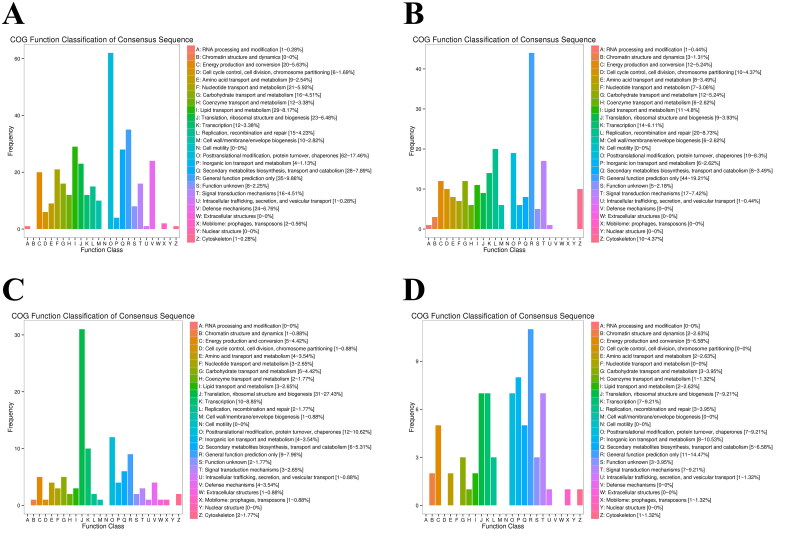
Using the estrus period as the control group, the enrichment of DEGs and DETs in the COG database of yak ovaries during the anestrus period was analyzed. (A) Enrichment level of DEGs up-regulated in COG in yaks during anestrus compared with estrus. (B) Enrichment level of DEGs down-regulated in COG in yaks during anestrus compared with estrus. (C) Enrichment level of DETs up-regulated in COG in yaks during anestrus compared with estrus. (D) Enrichment level of DETs down-regulated in COG in yaks during anestrus compared with estrus. COG, Clusters of Orthologous Groups; DEG, differentially expressed gene; DET, differentially expressed transcript.

The standardized gene function classification system (COG database) was used to classify homologous gene products. The results of COG analysis showed that compared with the estrus period, the up-regulated DEGs and DETs in the ovaries during the quiescent period were distributed in 19 and 24 categories, respectively (Figures 3A and C), while the down-regulated DEGs and DETs were distributed in 21 and 22 categories, respectively (Figures 3B and D). Among them, 76 up-regulated and 229 down-regulated DEGs were mainly related to “general function prediction” (44), “post-translational modification, protein turnover, molecular chaperone” (19) and “replication, recombination and repair” (20), and down-regulation was the main trend. Among the 113 up-regulated DETs, most were related to “post-translational modification, protein turnover, molecular chaperone” (12) and “transcription” (10). The 335 downregulated DETs were mainly enriched in the fields of “post-translational modification, protein turnover and molecular chaperones” (60), “general function prediction” (35), “lipid transport and metabolism” (29) and “biosynthesis, transport and catabolism of secondary metabolites” (28), with downregulation as the main trend.

### Validation of selected DEGs by qRT-PCR

Genes with different expression levels in energy metabolism, rhythm, and reproductive endocrine processes were screened out and verified by qRT-PCR. *SIRT1*, *LCHGR*, *INSL3*, and *ZP2* genes were significantly up-regulated in yak ovaries during quiestrus. On the other hand, *CSNK1A1*, *BMP15*, *ClocK*, *BMAL1*, *SLIT3*, *KISS1*, and *INHBA* gene expression in the static stage of yak ovarian significantly lower. These results are consistent with data from RNA-seq measurements (Table 1).

**Table 1.** Validation of selected DEGs by qRT-PCR analysis.

Gene ID	Gene name	qRT-PCR		ONT log_2_^FC^	Regulated
		log_2_^FC^	Regulated		
102284455	CSNK1A1	−3.89	Down	−3.91	Down
102279085	BMP15	−5.14	Down	−5.11	Down
102274673	CLOCK	−3.45	Down	−3.43	Down
102272853	BMAL1	−1.33	Down	−1.30	Down
102283723	SLIT3	−1.10	Down	−1.09	Down
102265082	KISS1	−1.24	Down	−1.23	Down
102277358	INHBA	−1.43	Down	−1.40	Down
102277382	SIRT1	2.36	Up	2.35	Up
102282677	LCHGR	2.21	Up	2.24	Up
102281340	INSL3	2.25	Up	2.22	Up
102281906	ZP2	4.67	Up	4.66	Up

DEG, diferentially expressed gene; qRT-PCR, quantitative real-time polymerase chain reaction.

## Discussion

### Regulation of reproductive status by energy metabolism

Mammalian reproduction is highly dependent on the successful regulation of energy metabolism (19). However, the disruption of the physiological balance between reproductive function and energy metabolism can lead to a series of reproductive disorders, such as prolonged anestrus, infertility, and sterility (20). Yaks live in an extremely harsh environment on the plateau, with insufficient food supply, resulting in nutrient deficiency and energy imbalance (21). In order to maintain basic energy metabolism needs, the body reduces or blocks energy from the reproductive system, thus affecting the normal reproductive cycle of yaks (22). The body regulates estrus and anestrus through different signaling molecules (hormones, neuropeptides, and metabolites) by regulating signaling pathways related to energy metabolism, including mTOR (23), AMPK (24), and sirtuin1 (SIRT1)(25). KEGG enrichment analysis found that during the anestrus period, the expression levels of genes related to oxidative phosphorylation and glutathione metabolism were downregulated, indicating that the energy metabolism of yaks was negative, which hindered the normal development of follicles and reduced the development and fertility of oocytes, which may be related to ovarian cell autophagy (26). Autophagy is a process of selective degradation and recycling of intracellular components. It plays a vital role in maintaining cellular homeostasis and cell survival by engulfing damaged and harmful proteins, lipids, and organelles. Autophagy is triggered by nutrient deficiency (27). SIRT1 is a multifunctional enzyme that is dependent on nicotinamide adenine dinucleotide and plays a key role in the regulation of cellular autophagy (28). This study found that SIRT1 expression was downregulated in the ovaries of yaks during the anestrus period, leading to dysregulation of ovarian cell autophagy, blocked oocyte maturation, and blocked reproductive cycle. Autophagy is also regulated by PI3K-Akt-mTOR and AMPK (29–31), which was also confirmed by the results of this study. KEGG analysis results showed that genes related to the PI3K-Akt-mTOR and AMPK signaling pathways were either downregulated or upregulated during the anestrus period. A negative energy balance can also reduce the secretion of reproductive hormones, ultimately leading to a prolonged anestrus period (32). Of course, postpartum lactation can also cause or aggravate negative energy balance in yaks, leading to long-term anestrus. Early weaning is a suitable strategy to improve the reproductive status of yaks after childbirth and enable them to quickly enter the next estrus cycle (33). Therefore, providing additional nutrients during cold seasons, food shortages, and during the pre- and postpartum periods is particularly important to ensure the normal estrus cycle of yaks (34).

### Circadian rhythms regulate yak reproduction

DEGs and DETs analysis showed that *LCHGR*, *INSL3*, and *ZP2* genes were upregulated, whereas *casein kinase 1 isoform alpha1* (*CSNK1A1*), *SLIT3*, *INHBA*, *KISS1*, *clock circadian regulator* (*ClocK*), and *basic helix-loop-helix ARNT-like protein 1* (*Bmal1*) were downregulated during the quiescent period, suggesting possible involvement in circadian rhythms. Natural periodic photoperiod plays an important role in mammalian reproduction. The 24-hour rhythm generated by the circadian clock is integrated into feedback regulatory loops and repair mechanisms to maintain homeostasis. Regulation of the reproductive system is highly integrated with circadian rhythms (35). Melatonin is mainly produced by the pineal gland and regulates changes in neuroendocrine function of the hypothalamic-pituitary-gonadal (HPG) axis, synchronizing the reproductive cycle with the light cycle in various animals (36). The reproductive process of yak is also strictly regulated by the circadian clock system (37, 38), which plays an important role in regulating the production and secretion of sex hormones, follicle development and maturation, ovulation, and estrus in female mammals (39, 40). Bmal1 and ClocK are key regulators of the circadian clock system. Knocking down the *Bmal1* gene in porcine ovarian granulosa cells cultured in vitro significantly reduced the mRNA and protein expression levels of the steroidogenesis regulatory protein Star, while gene silencing inhibited the synthesis of progesterone and estradiol (41). In mouse models, knockout of *ClocK* significantly reduces fertility through oocyte arrest and reduces litter size (42). Furthermore, exposure to slowly alternating light decreased core body temperature and melatonin release, reduced rest consolidation, and prolonged total rest time in pregnant cows, whereas lower light levels reduced rest time. However, exposure to chronic alternating light prior to calving increased milk production and milk fat content. Thus, small changes to the circadian clock system can stabilize the internal environment, support lactation, and ensure the next estrus cycle (43). Other studies have shown that exposure to different colors of light can affect hormone secretion, growth, and development in dairy cows. For example, exposure to yellow light increases water intake, feed intake, rumination time, and weight gain, while exposure to blue light reduces melatonin secretion, which can have a negative impact on the development of dairy cows (44). This study found that *ClocK* and *Bmal1* were downregulated during the dormant period, resulting in a decrease in preovulatory sex hormone levels, stagnation of ovarian follicle development, and ovulation blockage in yaks. Therefore, we speculate that the expression of *CSNK1A1*, *SLIT3*, *INHBA*, *KISS1*, *ClocK*, and *Bmal1* is upregulated during the rhythmic light cycle from the summer solstice to the winter solstice. However, the signaling pathways that regulate the yak circadian clock system and affect postpartum estrus through the HPG axis are still unclear.

### Hormone regulation of the reproduction cycle

GnRH plays an important regulatory role in the reproductive process of mammals by controlling the synthesis and release of gonadotropin (45). Recent studies have shown that GnRH binds to the kisspeptin 1 (KISS1) receptor to activate the HPG axis, thereby regulating follicle development, oocyte maturation, and estrus ovulation (46, 47). KISS1 gene mutations and subsequent KISS1/KISS1R system dysregulation can lead to a range of idiopathic clinical symptoms, such as hypogonadotropic hypogonadism, central precocious puberty, and female infertility (48). This study found that during the resting period of yaks, *KISS1* gene expression was downregulated, suggesting that reduced *KISS1* expression inhibits the secretion of GnRH, thereby inducing the resting period of yaks. LH and FSH regulate estrus, pregnancy, and resting period in female mammal (49). Previous research reports have shown that hunger can increase the secretion of growth hormone-releasing peptide, thereby inhibiting the secretion of LH, slowing down or preventing the maturation of follicles, leading to insufficient progesterone secretion, causing infertility and miscarriage (50). Likewise, FSH plays a vital role in regulating ovarian endocrine function (51). This study found that down-regulating *BMP15* gene expression during the resting phase may inhibit the activation of FSH and lead to the arrest of follicle development during estrus. There are two forms of mammalian FSH: partially glycosylated FSH (FSH21/18), which is mainly secreted during the peak reproductive period; and fully glycosylated FSH (FSH24), which is the main form during perimenopause/menopause. Compared with FSH21/18, FSH24 can reduce follicle survival rate, cause basement membrane rupture, and even discharge oocytes, which is significantly detrimental to the binding and activation of FSH receptor (FSHR) (52–54). In the present study, the expression of *luteinizing hormone/chorionic gonadotropin receptor* (*LCHGR*) was upregulated during the quiescent period, indicating that high expression of *LHCGR* does not induce estrus and follicular development in yaks. Upregulated expression during the quiescent period but not during the estrus period suggests that *M91_03062* may induce the quiescent period. In addition, melatonin can inhibit the apoptosis and proliferation of sheep follicular cells (TCs) cultured in vitro and activate the PI3K/Akt signaling pathway, which regulates the synthesis and secretion of progesterone after TC ovulation. This finding is of great significance for improving follicular arrest and delaying ovarian aging (55). Excessive secretion of FSH and threshold secretion of progesterone by the HPG axis triggers negative feedback regulation, inhibiting the secretion of GnRH and FSH, thereby interfering with follicular development and preventing estrus in yaks (56).

## Conclusion

In this study, the DEGs and DETs in ovarian tissues of yaks during estrus and quiescence were identified using third-generation sequencing technology. The results showed that SIRT1, CSNK1A1, SLIT3, INHBA, INSL3, ZP2, Clock, BMP15, Bmal1, KISS1, and LCHGR regulated the postpartum quiescence and reproductive cycle of yaks; in addition, kisspeptin-G protein-coupled receptor, PI3K-Akt-mTOR, and AMPK signaling pathways also regulated ovarian function and reproductive cycle of yaks. The results of this study will help to clarify the molecular mechanism of ovarian function and provide a reference for further exploring the physiological characteristics of yaks during estrus and quiescence.

## Supplementary Material

Declaration of competing interest.docx

## Data Availability

The data that support the findings of this study are available from the corresponding author upon reasonable request.
