# Supplementary material for: Comparative analysis of differentially expressed genes and transcripts in the ovary of yak in estrus and anestrus
Source: Anim Biotechnol. 2024 Nov 18;35(1):2427757. doi: 10.1080/10495398.2024.2427757 (PMC12674260; doi:10.1080/10495398.2024.2427757)
Supplement: Declaration of competing interest.docx [file LABT_A_2427757_SM4433.docx]

**Declaration of competing interest**

None of the authors have any conflict of interest to declare.
